# Computational reassessment of RNA-seq data reveals key genes in active tuberculosis

**DOI:** 10.1371/journal.pone.0305582

**Published:** 2024-06-27

**Authors:** Rakesh Arya, Hemlata Shakya, Reetika Chaurasia, Surendra Kumar, Joseph M. Vinetz, Jong Joo Kim

**Affiliations:** 1 Department of Biotechnology, Yeungnam University, Gyeongsan, Gyeongbuk, South Korea; 2 Department of Biomedical Engineering, Shri G. S. Institute of Technology and Science, Indore, Madhya Pradesh, India; 3 Department of Internal Medicine, Section of Infectious Diseases, Yale University School of Medicine, New Haven, CT, United States of America; 4 Department of Orthopaedic Surgery, The Johns Hopkins University School of Medicine, Baltimore, MD, United States of America; Stellenbosch University, SOUTH AFRICA

## Abstract

**Background:**

Tuberculosis is a serious life-threatening disease among the top global health challenges and rapid and effective diagnostic biomarkers are vital for early diagnosis especially given the increasing prevalence of multidrug resistance.

**Methods:**

Two human whole blood microarray datasets, GSE42826 and GSE42830 were retrieved from publicly available gene expression omnibus (GEO) database. Deregulated genes (DEGs) were identified using GEO2R online tool and Gene Ontology (GO), protein-protein interaction (PPI) network analysis was performed using Metascape and STRING databases. Significant genes (n = 8) were identified using T-test/ANOVA and Molecular Complex Detection (MCODE) score ≥10, which was validated in GSE34608 dataset. The diagnostic potential of three biomarkers was assessed using Area Under Curve (AUC) of Receiver Operating Characteristic (ROC) plot. The transcriptional levels of these genes were also examined in a separate dataset GSE31348, to monitor the patterns of variation during tuberculosis treatment.

**Results:**

A total of 62 common DEGs (57 upregulated, 7 downregulated genes) were identified in two discovery datasets. GO functions and pathway enrichment analysis shed light on the functional roles of these DEGs in immune response and type-II interferon signaling. The genes in Module-1 (n = 18) were linked to innate immune response, interferon-gamma signaling. The common genes (n = 8) were validated in GSE34608 dataset, that corroborates the results obtained from discovery sets. The gene expression levels demonstrated responsiveness to *Mtb* infection during anti-TB therapy in GSE31348 dataset. In GSE34608 dataset, the expression levels of three specific genes, GBP5, IFITM3, and EPSTI1, emerged as potential diagnostic makers. In combination, these genes scored remarkable diagnostic performance with 100% sensitivity and 89% specificity, resulting in an impressive Area Under Curve (AUC) of 0.958. However, GBP5 alone showed the highest AUC of 0.986 with 100% sensitivity and 89% specificity.

**Conclusions:**

The study presents valuable insights into the critical gene network perturbed during tuberculosis. These genes are determinants for assessing the effectiveness of an anti-TB response and distinguishing between active TB and healthy individuals. GBP5, IFITM3 and EPSTI1 emerged as candidate core genes in TB and holds potential as novel molecular targets for the development of interventions in the treatment of TB.

## Introduction

Tuberculosis (TB), caused by *Mycobacterium tuberculosis* (*Mtb*), is one of the lethal infectious diseases with a high mortality rate. Globally, TB affects more than 10 million individuals, leading to approximately 1.6 million annual fatalities [1]. Around one-fourth of the world’s total population carries latent tuberculosis infections (LTBI), which can potentially progress to active tuberculosis infections (ATBI) during their lifetime [2]. The risk of progression increases when the immune system is compromised due to concurrent conditions like HIV-coinfection, diabetes, and organ transplantation [2, 3]. *Mtb* is often resistant to one or more antituberculous drugs, determination of which is challenging in the resource-limited setting. Early identification of lack of response to therapy using novel biomarkers is an important goal of clinical tuberculosis care.

Current diagnostic methods such as chest X-ray, acid-fast bacilli (AFB) staining, solid/liquid cultures, and nucleic acid amplification tests such as GeneXpert *MTB*/RIF, are carried out in response to symptoms and are laboratory-based [4]. Other tests that assess the immune response to *Mtb* antigens such as tuberculin skin tests (TST), and interferon-gamma release assays (IGRAs) come with drawbacks such as time, expense, potential cross-reactivity, and limited sensitivity [5]. These tests cannot distinguish different clinical manifestations of tuberculosis. Therefore, rapid and effective diagnostics approaches are needed to identify novel biomarkers in various body fluids such as blood/serum, urine, sputum, and bronchoalveolar lavage (BAL) fluid, to differentiate among different categories of TB and specially to monitor early responses to treatment.

Samples obtained from TB patients, including blood/serum, sputum, saliva, and urine contain both host and pathogen biomarkers [6]. Biomarkers derived from pathogens in the blood/serum show exceptional specificity for diagnosing tuberculosis. Comparison to host-related proteins, pathogens-derived proteins, non-peptide antigens (lipids, carbohydrates), or peptides is present in lower quantities and often near analytical methods’s lower limit of detection (for example, <10 ppM). Because of low-abundance peptides, powerful techniques like mass spectrometry, specifically liquid chromatography coupled with tandem mass spectrometry LC-MS/MS, can be employed for the identification of potential diagnostic biomarkers, as demonstrated by Kruh-Garcia et al. [7]. Several ‘omics’ approaches, including genomics, transcriptomics, metabolomics, and proteomics, have advanced the use of high-throughput methods/instruments for rapid and accurate analysis of comprehensive expression changes during tuberculosis infection [8]. The progression of TB disease may be correlated with the proteomics patterns of proteins released by *Mtb* and those produced by the normal lung flora. High-throughput techniques, including Next-Generation Sequencing (NGS) and Microarray, are employed to conduct transcriptome profiling, aiming to pinpoint the genes that exhibit differential expression in human diseases like tuberculosis.

Microarray gene chips can generate large amounts of data which can be re-analyzed to identify the deregulated biosignatures and related functional pathways [9]. However, the results often exhibit inconsistency due to the constraints of conducting studies with a single population and the inherent variability in the samples. Therefore, expression profiling data from several different studies can be combined which can offer insights into the quest for discovery biomarkers.

In the present study, we reanalyzed two publicly accessible microarray datasets, namely GSE342826 and GSE42830. These datasets encompass a total of 27 individuals diagnosed with tuberculosis (TB) and 90 individuals serving as healthy controls, all of whom contributed whole blood samples. We employed various online tools and computation approaches to detect genes that exhibit deregulation (referred to as “deregulated genes” or DEGs). To further elucidate the functional implications of these DEGs in tuberculosis, we performed Gene Ontology (GO) pathway analysis and protein-protein interaction (PPI) network analysis. This comprehensive analysis allowed us to delineate the roles and associations of the key deregulated genes in tuberculosis. These findings have significant implications for the identification of potential biomarkers for diagnosing tuberculosis. These findings hold promise for their application in anti-TB diagnosis and assessing response therapy, potentially aiding in different phases of treatment.

## Methods

### Acquisition of gene expression microarray data from NCBI GEO

The NCBI Gene Expression Omnibus database (GEO, https://www.ncbi.nlm.nih.gov/geo) is a freely accessible public repository that stores and shares high-throughput functional genomics data, including microarray and next-generation sequencing. In this study, we obtained four microarray datasets from the GEO database by searching for ’tuberculosis, control’. Considering previous studies on blood-based sample diagnostics for tuberculosis, we inferred that the sample sizes in all four datasets, comprising both tuberculosis and control samples, were suitable for our analysis. The R/Bioconductor package (*ver*. 4.3.0), GEOquery was used to extract the gene expression data from two discovery datasets GSE42826 and GSE42830 [10] and two validation datasets GSE34608 and GSE31348. GSE42826 consists of 52 control and 11 TB samples (GPL10558 platform; Illumina HumanHT-12 V4.0 expression bead chip). The GSE42830 dataset consists of 38 control and 16 TB samples (GPL10558 platform; Illumina HumanHT-12 V4.0 expression bead chip). GSE34608 and GSE31348 datasets were used for the validation of important genes. GSE34608 consists of 18 controls and 8 TB samples (GPL6480 platform; Agilent-014850 Whole Human Genome Microarray 4x44K G4112F) [11]. GSE31348 consists of 27 subject samples at five-time points (135 samples): diagnosis, treatment for 1, 2, 4, and 26 weeks (GPL570 platform; Affymetrix Human Genome U133 Plus 2.0 Array) (Cliff et al., 2013) [12]. All the data from the GEO database was accessed on 22 September 2023. The data has been taken from public database, so IRB or ethical committee or informed consent statements are not required.

### Identification of deregulated genes (DEGs) between TB and control groups

The 4 datasets were downloaded as raw data matrix files with microarray platform annotations from NCBI. These data were analyzed using an online tool, MetaboAnalyst (*ver*. 5.0; https://www.metaboanalyst.ca/), heatmaps and Principal Component Analysis (PCA) plots were generated after selecting the normalization type as quantile normalization and data scaling as auto-scaling [13]. Gene expression data were analyzed with the inbuilt tool, GEO2R (https://www.ncbi.nlm.nih.gov/geo/geo2r/) for identifying deregulated genes (DEGs) between TB and control groups [14]. The *p*-value adjustment of Benjamini & Hochberg (False discovery rate) was used. The data transformation was set to automatic mode, limma precision weights (vooma) were applied along with data normalization. The rows with missing corresponding gene symbol information were omitted from the analysis. The filtering criteria of log_2_ (fold change) ≥ ±1 and *p-value < 0*.*01* were followed for the selection of statistically significant DEGs from both GSE42826 and GSE42830 datasets. The common up- or down-regulated DEGs from both datasets were represented by a Venn diagram.

### Gene Ontology (GO) and pathway enrichment analyses of DEGs

Metascape (https://www.metascape.org/) is an online portal that provides comprehensive gene list annotations and biological information about genes and proteins [15]. The function of these DEGs was investigated using Gene Oncology (GO) of biological processes (BP), Reactome, and WikiPathways enrichment analysis using Metascape. The network analysis of enriched terms was performed where the nodes that share the same cluster ID and are typically close to each other are colored by cluster ID, and the nodes where terms containing more genes tend to have a more significant *p*-value are colored by *p*-value.

### Protein-protein interaction (PPI) network and Module analysis

The PPI network was prepared using the online database, Search Tool for the Retrieval of Interacting Genes (STRING, *ver*. 12; https://www.string-db.org) [16]. The minimum interaction score required was set at medium confidence of 0.4. The PPI network was further visualized and analyzed with an open-source software platform, Cytoscape (*ver*. 3.10.1). The Molecular Complex Detection (MCODE) app embedded in the Cytoscape environment was used for clustering a given network based on topology to find highly interconnected regions as Modules. The GO functions (biological processes and molecular functions), pathways (WikiPathways, KEGG, Reactome), and protein domains (PFAM, INTERPRO) of Module-1 were analyzed using Metascape and STRING.

### Validation of important genes and ROC analysis

The significant genes were selected based on two criteria of common top 8 proteins in the heatmap analysis by T-test/ANOVA in MetaboAnalyst and MCODE score ≥10. The expression levels of important genes were validated in independent datasets GSE34608 and GSE31348 and shown as boxplots with interquartile ranges. By using data mining and validation by clinical study approaches, the geometric mean of the three gene transcription levels is defined as the TB score [TBscore = (∛GBP5*IFITM3*EPSTI1)] [17]. The TBscore and individual genes were tested for diagnostic potential by Receiver Operating Characteristic (ROC) curves using an online web tool, SRPLOT (https://www.bioinformatics.com.cn/).

## Results

### Identification of deregulated genes between TB and control groups

**Fig 1:**
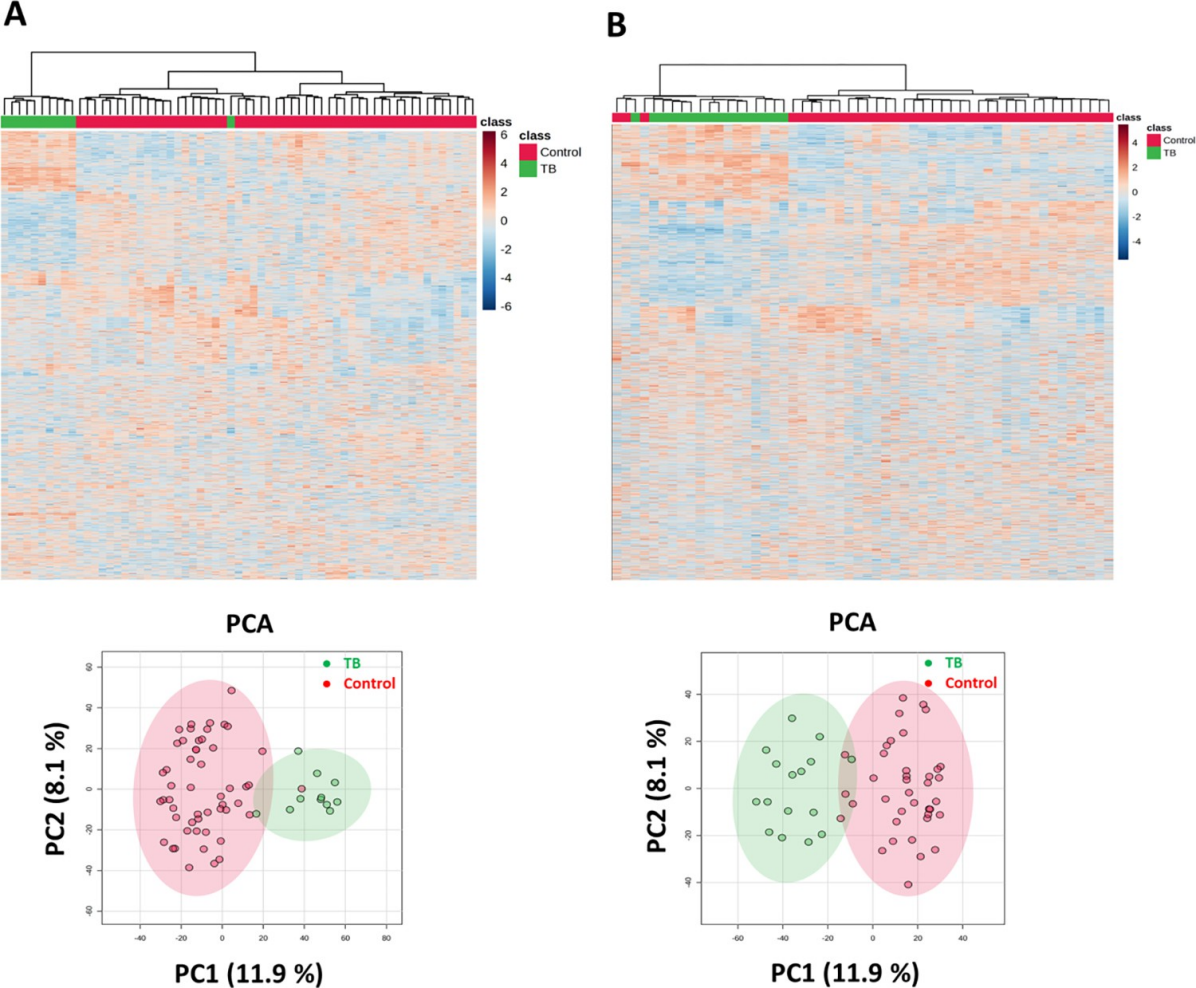
Gene expression levels of TB and control groups using MetaboAnalyst. (A) Heatmap and PCA plot of GSE42826 dataset; (B) Heatmap and PCA plot of GSE42830 dataset.

Both the gene expression data, GSE42826 and GSE42830 showed 31266 features individually. In GSE42826, there were 52 control samples and 11 samples from patients with TB, while GSE42830 had 38 control samples and 16 TB patients’ samples. Both datasets showed distinct separation between the control and TB sample groups, as indicated by the heatmaps and PCA plots (Fig 1).

**Fig 2:**
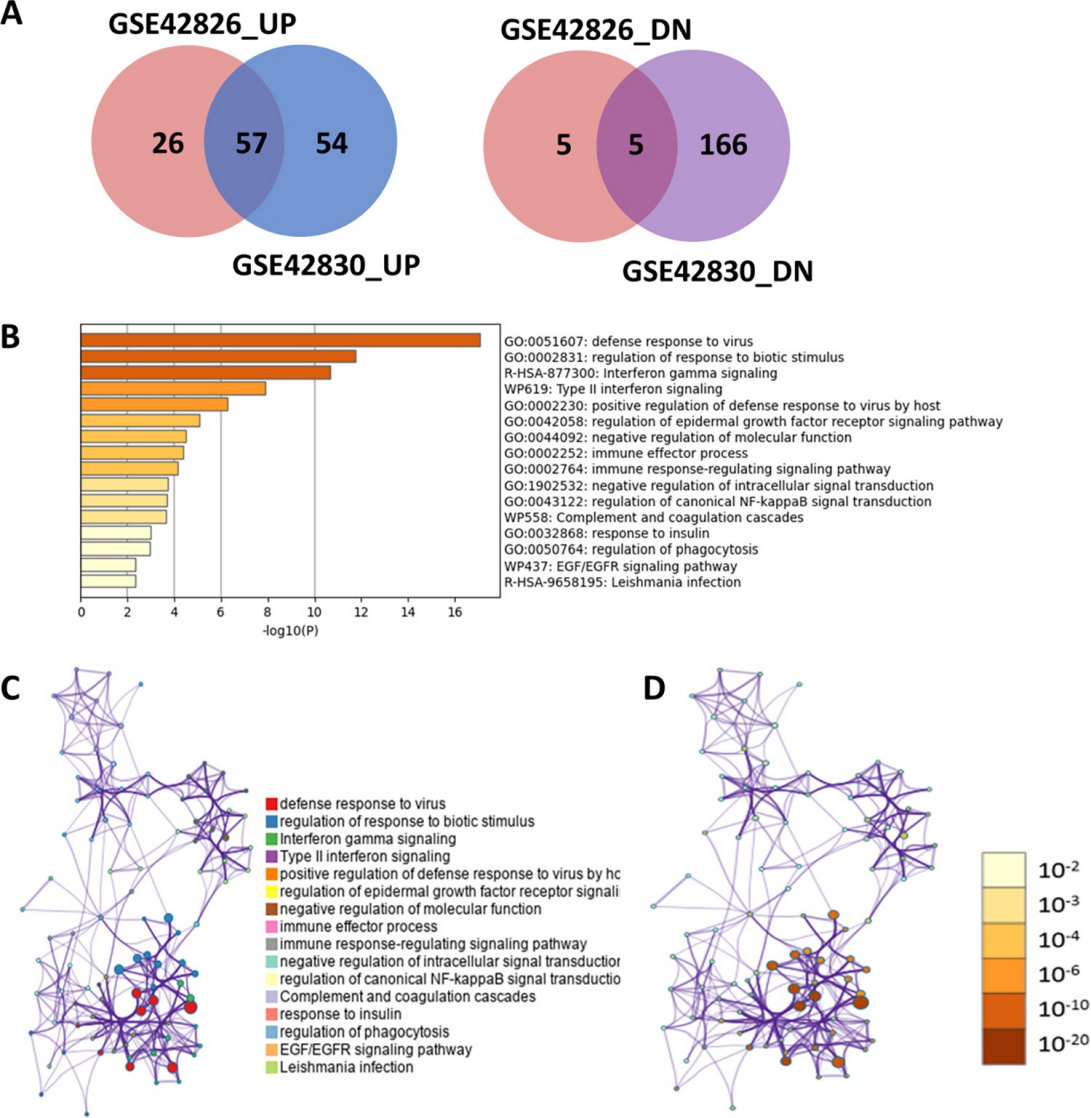
Selection and function of deregulated genes (DEGs). (A) The Venn diagram illustrates DEGs identified in two datasets: GSE42826 and GSE42830. In total, 57 were found to be upregulated, while 5 genes were downregulated, and these were shared between both GSE datasets. (B) The functional annotation of DEGs was conducted using Metascape online tool, resulting in the representation of the top 16 terms in a bar plot based on their p-value (log_10_ scale). (C) The Network of enriched terms is color-coded by cluster ID, a configuration where nodes sharing the same cluster ID tend to be closely positioned to one another; (D) In this network, the terms are color-coded based on their p-value, with terms containing more associated genes having more significant *p*-value.

Utilizing GEO2R analysis, we identified 113 differentially expressed genes (DEGs) in GSE42826, 102 genes upregulated, and 11 genes downregulated. In GSE42826, there were 324 DEGs, containing 138 upregulated and 186 downregulated genes (Fig 2A). A Venn diagram revealed that among the 62 common DEGs, 57 genes exhibited upregulation, while 5 genes were downregulated in both datasets (Fig 2A).

### Gene Ontology (GO) and pathway enrichment analyses of DEGs

Metascape was employed to conduct Gene Ontology (GO) function and pathway enrichment analyses on the identified DEGs. The results showed a notable enrichment of DEGs associated with various processes, including response to biotic stimulus, interferon gamma signaling, Type-II interferon signaling, immune response-regulating signaling pathway, complement and coagulation cascades (Fig 2B). Using Metascape, the subclass of representative terms from gene function analysis was transformed into a network arrangement (Fig 2C). Additionally, a tree-based hierarchical clustering method, based on Kappa-statistical similarities, was employed to group the important terms derived from the gene function analysis. Each term was represented by a circle node, and the size of the node depended on how many input genes fell under that term. Terms with a kappa score > 0.3 are linked by an edge. A network of enriched terms colored by cluster ID, where nodes with the same cluster ID are situated close to one another (Fig 2C). Furthermore, the network was colored by p-value, highlighting that terms encompassing more genes tended to have more significant p-values (Fig 2D).

### Protein-protein interaction network and Module analysis

The PPI (Protein-Protein Interaction) network analysis was conducted on 62 common DEGs using STRING, followed by an in-depth examination using the MCODE app within the Cytoscape environment. The analysis yielded two modules, Module-1 consisting of 18 genes, representing a core set of functional genes, and Module 2 with 3 genes (S1 Table). Gene Ontology (GO) analysis of these 18 genes revealed their involvement in a range of functions and pathways, including interferon-gamma signaling, and immune response to tuberculosis (S1 Fig). The STRING network analysis of these 18 genes within Module 1 showed that differentially expressed genes are involved in known and predicted protein-protein interactions supported by a high confidence score > 0.4. The predicted associations among all genes are designated by seven different types of colors as fusion, neighborhood, co-occurrence, experimental, text mining, database, and co-expression evidences (S1 Fig). Examining the expression levels of these 18 genes in the GSE42826 dataset, it was evident that they were significantly upregulated in tuberculosis patients, as represented in the heatmap (S1 Fig). This consistency in gene expression levels was also observed in GSE42830, as indicated by heatmap analysis (S1 Fig). Moreover, the analysis of GO, pathways, and protein domains highlights that the GO functions were notably associated with biological processes such as defense response and innate immune response, as well as molecular functions like identical protein binding. Pathway analysis, including Wiki, KEGG, and Reactome pathways indicated that these 18 genes were involved in processes like Type-II interferon signaling, immune response to tuberculosis, NOD-like receptor signaling pathway, and interferon-gamma signaling. PFAM and INTERPRO protein domain analysis identified N-terminal and C-terminal domains in Guanylate-binding protein as significant features (Table 1).

**Table 1:** Functional analysis of 18 genes in Module-1 using STRING database.

Term	Description	Count	FDR
**Biological Processes (GO**)			
GO:0098542	Defense response to other organism	14 of 989	2.82E-11
GO:0006952	Defense response	15 of 1394	3.14E-11
GO:0051607	Defense response to virus	10 of 252	3.14E-11
GO:0006955	Immune response	14 of 1321	2.96E-10
GO:0045087	Innate immune response	12 of 754	3.19E-10
**Molecular Function (GO)**			
GO:0042802	Identical protein binding	10 of 2144	0.0224
**Wiki Pathways**			
WP619	Type II interferon signaling	5 of 37	2.25E-07
WP4197	Immune response to tuberculosis	4 of 23	3.29E-06
**KEGG Pathways**			
hsa04621	NOD-like receptor signaling pathway	4 of 173	0.0058
**Reactome Pathways**			
HSA-913531	Interferon Signaling	11 of 199	1.05E-14
HSA-909733	Interferon alpha/beta signaling	7 of 71	4.04E-10
HSA-877300	Interferon gamma signaling	6 of 90	1.15E-07
HSA-168256	Immune System	12 of 1979	5.16E-06
HSA-1169410	Antiviral mechanism by IFN-stimulated genes	3 of 82	0.0229
**PFAM Protein Domains**			
PF02263	Guanylate-binding protein, N-terminal domain	3 of 11	0.00038
PF02841	Guanylate-binding protein, C-terminal domain	3 of 9	0.00038
**INTERPRO Protein Domains and Features**			
IPR003191	Guanylate-binding protein/Atlastin, C-terminal	3 of 9	0.00055
IPR015894	Guanylate-binding protein, N-terminal	3 of 11	0.00055
IPR030386	GB1/RHD3-type guanine nucleotide-binding (G) domain	3 of 11	0.00055
IPR036543	Guanylate-binding protein, C-terminal domain superfamily	3 of 10	0.00055
IPR037684	Guanylate-binding protein, C-terminal	3 of 7	0.00055

### Statistically important gene analysis

**Fig 3:**
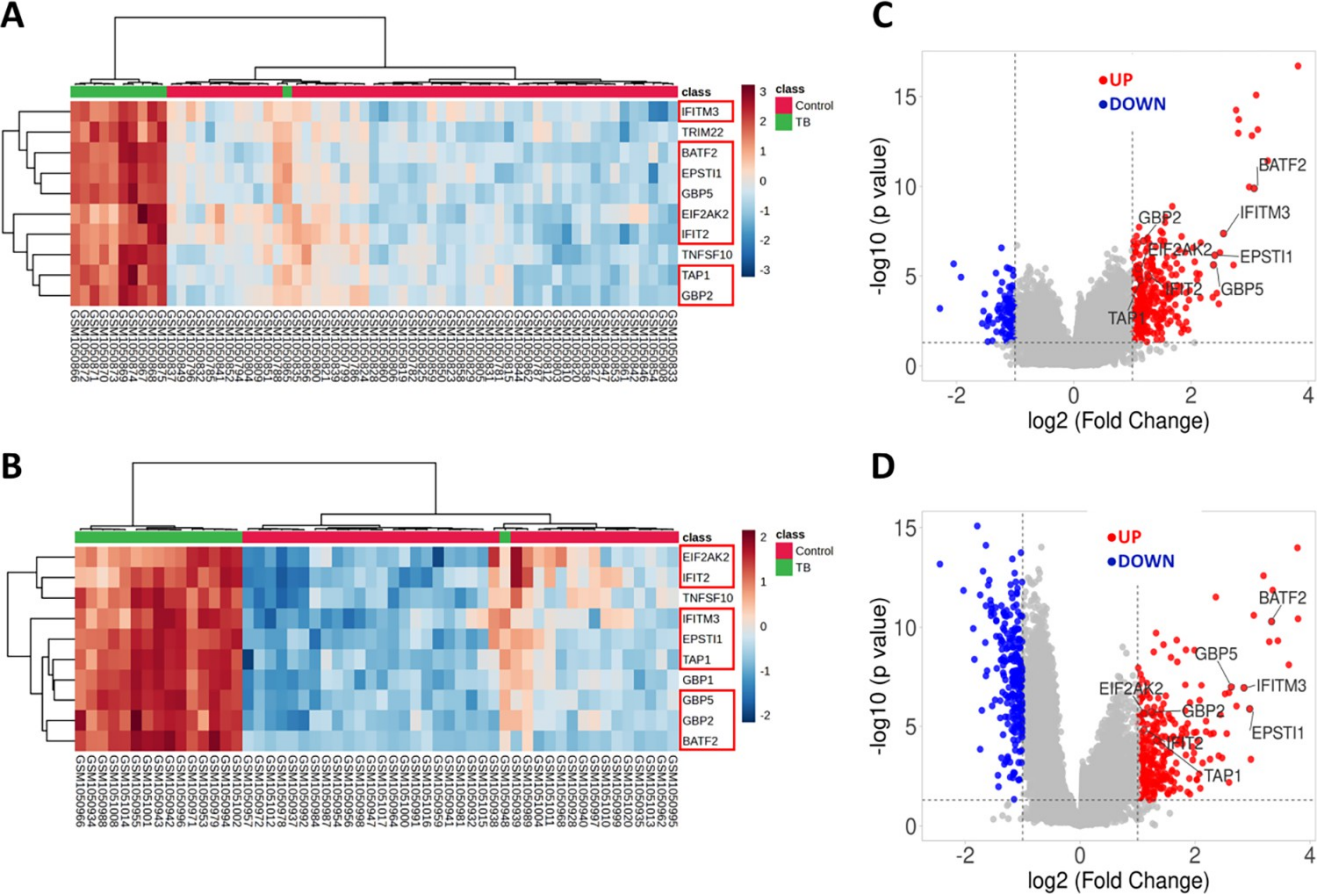
Selection of 8 important common deregulated genes (DEGs) in both datasets. Heatmaps showing 8 important genes selected based on T-test/ANOVA and MCODE score ≥10 (A) GSE42826 dataset; (B) GSE42830 dataset. In addition, the volcano plot depicts the differential gene expression in both datasets, employing a cutoff criterion of log_2_ (fold change) ≥ 1 and *p*-value < 0.01 (C) Volcano plot for the GSE42826 dataset; (D) Volcano plot for the GSE42830 dataset.

Eight prominent genes, namely (EIF2AK2, GBP5, GBP2, IFIT2, IFITM3, EPSTI1, BATF2, and TAP1) were selected as crucial genes from both datasets. They were chosen based on specific criteria, and their significance was further confirmed through heatmap analysis employing T-test/ANOVA in MetaboAnalyst, as well as an MCODE score ≥10 (Fig 3A and 3B, S2 Table). The volcano plots provided a clear representation of these eight vital genes, demonstrating their upregulation in both the discovery datasets (Fig 3C and 3D). Notably, these important genes were found to be significantly associated with interferon-gamma signaling pathways and the innate immune response as retrieved by the UniProt database (S2 Table).

### Validation and detection of gene expression during TB treatment

**Fig 4:**
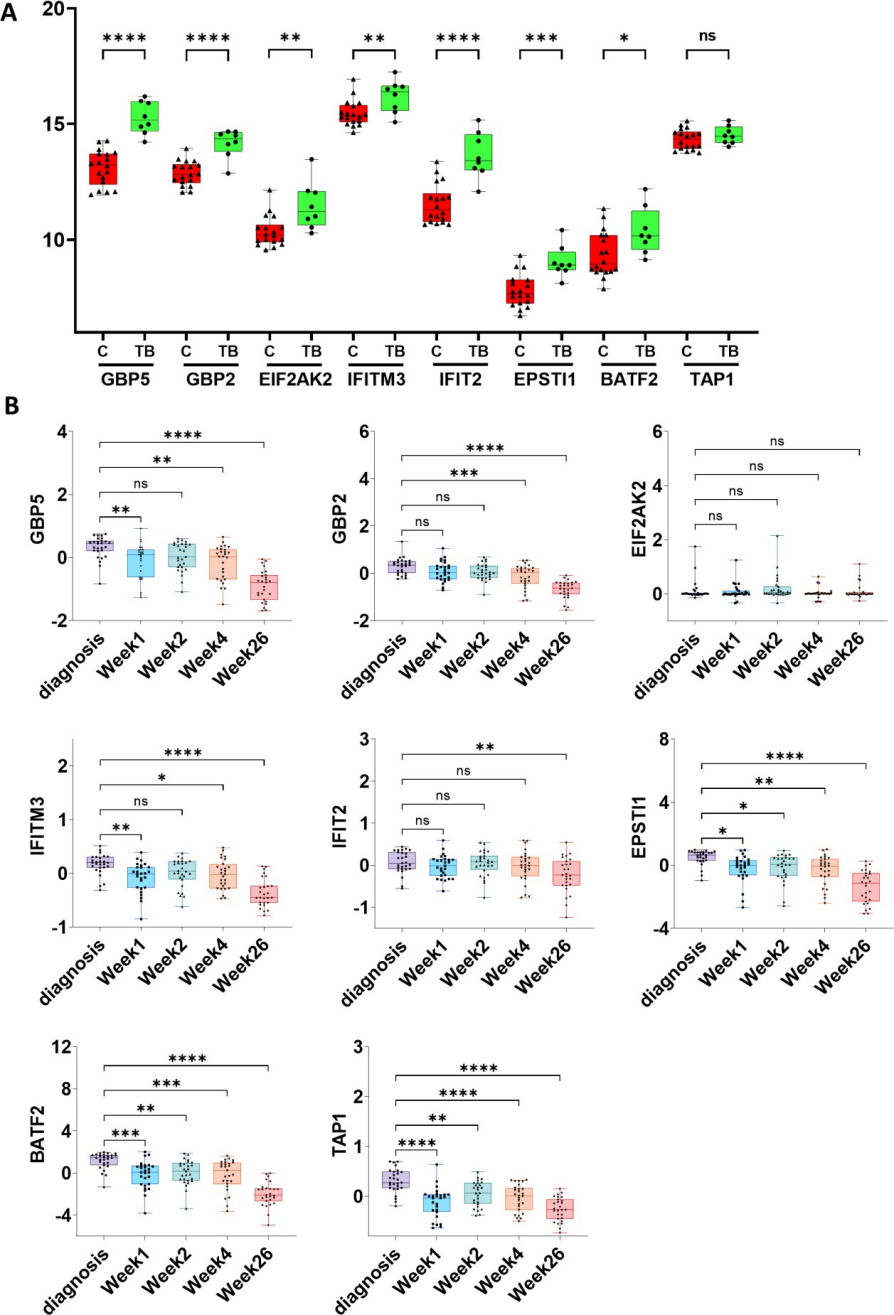
Expression validation of 8 important genes in GSE34608 and GSE31348 datasets. The boxplots show the expression level of GBP5, GBP2, EIF2AK2, IFITM3, IFIT2, EPSTI1, BATF2 and TAP1 genes during TB infection. (A) The boxplots depict the GSE34608 dataset; (B) The boxplots depict the GSE31348 dataset during TB treatment at five distinct time points. In each boxplot, the central horizontal line represents the median, while the ends represent the first quartile [Q1] and third quartile [Q3] defining the interquartile range (IQR). The ends of the central vertical line denote the minimum and maximum values. C = Control, TB = Tuberculosis, *p-value < 0.05, **p-value < 0.01, **p-*value* < 0.001, ****p-value < 0.0001, ns = non-significant results.

The validation of gene expression in the GSE34608 dataset revealed significant differences in the expression level of all 8 genes between the control and TB patient groups, with the exception TAP1 (Fig 4A). To explore how the expression level changes during the TB treatment regimen, we examined another validation dataset, GSE31348, which included a total of 135 samples from 27 TB patients at five different time points: diagnosis, treatment for 1, 2, 4, and 26 weeks. Heatmap analysis illustrated that the expression levels of most of the genes were down-regulated during TB treatment (S2 Fig). Among these 8 genes, GBP5, IFITM3, and EPSTI1 showed a significant decrease in expression level during the TB treatment (Fig 4B). This suggests that the three-gene panel could potentially serve as a valuable drug target for TB diagnosis.

### Diagnostic performance of significant genes

**Fig 5:**
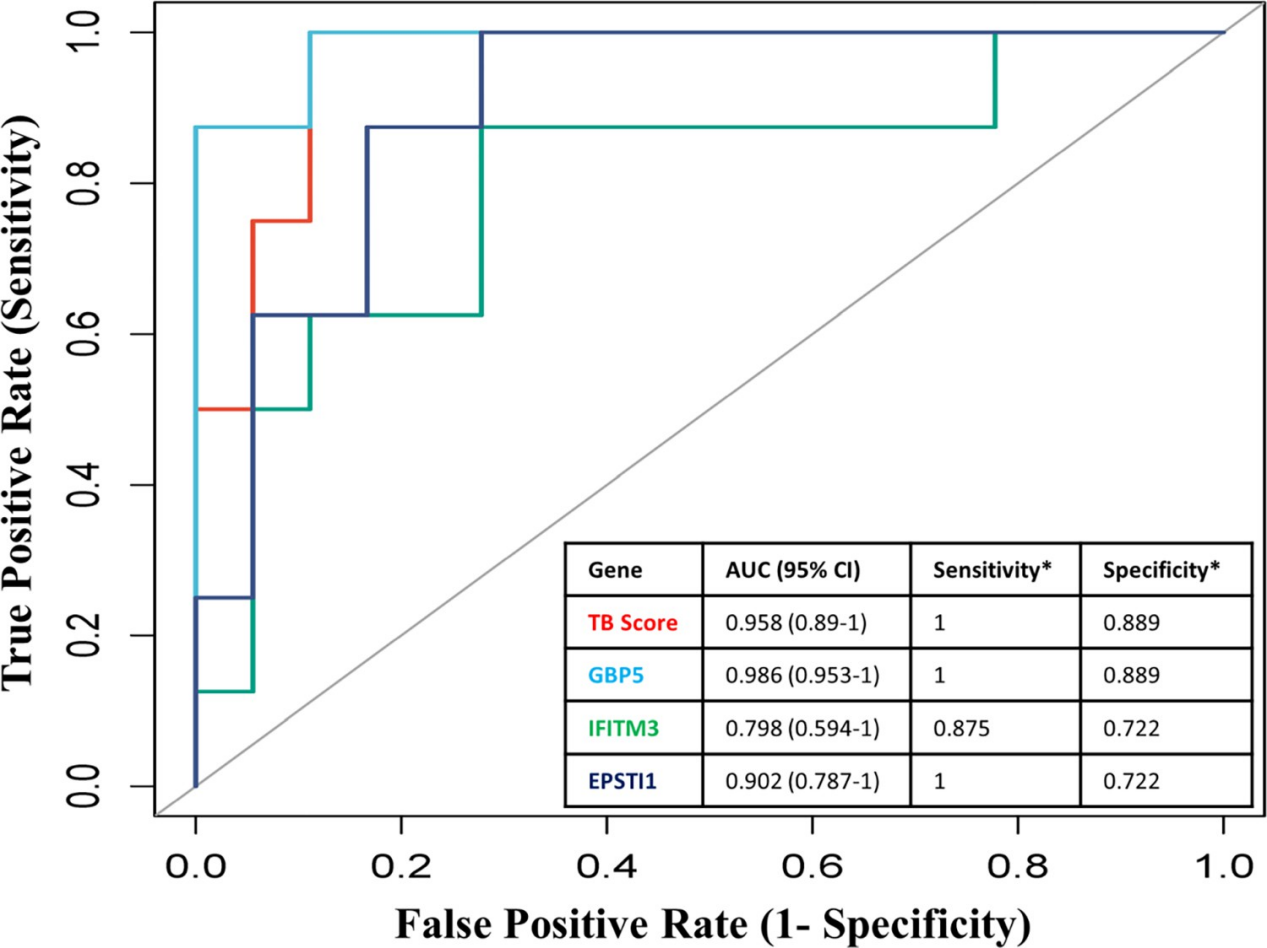
Performance of 3 genes by using ROC curve and the difference between the ROC curve of each gene and TBscore. (*based on Youden’s index).

From the pool of 8 important genes, we conducted a diagnostic assessment of three specific genes, GBP5, IFITM3, and EPSTI1, employing ROC_(AUC)_ curve analysis. These genes showed substantial variations between the TB and control groups in the GSE34608 validation dataset, which corroborates with the discovery dataset. To ascertain the sensitivity and specificity of these three genes, we assessed the TBscore in an independent gene expression validation dataset, GSE34608. The results revealed that GBP5 had a relatively higher AUC (0.986) compared to the combination TBscore which had an AUC of 0.958. However, IFITM3 (AUC 0.798) and EPSTI1 (AUC 0.902) showed lower AUC values when compared to the combination. It is also worth noting that the ROC curves of every gene differed significantly from those of TBscore combination, with a *p-value <0*.*05*, as outlined by Venkatraman [18]. The combined TBscore met all criteria effectively, with an AUC of 0.958 (95% CI 0.89–1), achieving a sensitivity of 100% and specificity of 89%. On the other hand, GBP5 alone demonstrated a sensitivity of 100% and a specificity of 89%, with a higher AUC of 0.986 compared to the TBscore. Therefore, GBP5 possesses the potential to effectively distinguish TB patients from control individuals (Fig 5).

## Discussion

The 2022 Global Tuberculosis Report by the World Health Organization (WHO) indicates that 1.6 million individuals died from tuberculosis worldwide in 2021 [1]. The global challenge of tuberculosis is exacerbated by the expense and availability of effective diagnostic techniques and treatments. To address this issue, studying the immune system’s defense mechanisms combating *Mtb* can provide insights into the development of innovative diagnostic and therapeutic approaches. Recent studies have shed light on the role of the innate immune response system, which serves as the first defense and possesses the ability to recognize foreign pathogenic bacterial antigens [19].

In the current study, we studied two GEO datasets, GSE42826 and GSE42830, to pinpoint key genes that undergo deregulation during tuberculosis infection. Both datasets demonstrated a robust separation between TB-affected and control groups by the heatmap and PCA plot (Fig 1). In total, we identified 62 differentially expressed genes (DEGs) through a combined analysis of these two GEO datasets (Fig 2). By performing a protein-protein interaction network analysis using the STRING database and MCODE analysis, we identified two modules: Module-1 encompasses 18 genes, including PLSCR1, STAT1, TRIM22, SAMD9L, BATF2, GBP5, GBP1, IFIT3, PARP9, IFI35, TAP1, GBP2, IFIT2, EPSTI1, OASL, TNFSF10, IFITM3, EIF2AK2, while Module-2 consists of 3 genes: FCGR1A, FCGR1B, and SERPING1 (S1 Table). To delve deeper into the functionality of these 18 genes within Module-1, we conducted a comprehensive functional and pathway analysis using the STRING database. This analysis revealed their involvement in critical processes such as the innate immune response to tuberculosis, interferon-gamma signaling, and the NOD-like receptor signaling pathway (Fig 2 and Table 1). Subsequent data analysis, employing STRING, T-Test/ANOVA in heatmap analysis, and MCODE score cutoff of ≥10, enabled us to pinpoint 8 important differentially expressed genes; GBP5, GBP2, EIF2AK2, IFITM3, IFIT2, EPSTI1, BATF2, and TAP1 (Fig 3 and S2 Table). These genes play essential roles in biological functions and pathways associated with interferon-gamma signaling and immune response to pathogens (S2 Table). The identification and deregulation of these important genes demonstrates a significant correlation with TB infection, as well as with the impact of anti-TB therapy at various treatment stages (Fig 4).

The three candidate genes, namely GBP5, IFITM3, and EPSTI1, consistently showed significant deregulation in both the GSE34608 and GSE31348 datasets, warranting their evaluation for diagnostic efficiency through ROC curve analysis. These genes showed a gradual reduction in expression levels with effective treatment, making them potential biomarkers for monitoring treatment effectiveness. The ROC analysis was conducted using the GSE34608 dataset, revealing that the combination of the three genes, referred to as “TBscore”, achieved an impressive sensitivity and specificity of 100% and 89% respectively with an AUC of 0.958. However, when considered individually, IFITM3 and EPSTI1 showed lower AUC of 0.798, (sensitivity- 87.5% and specificity- 72.2%) and 0.902 (sensitivity- 100% and specificity- 72.2%) respectively. In contrast, GBP5 stood out with the highest AUC of 0.986 when evaluated on its own, along with sensitivity and specificity of 100% and 88.9% respectively. The diagnostic potency of GBP5 surpasses that of the combined three-gene set. Consequently, GBP5 emerges as a promising biomarker for clinical tuberculosis diagnosis and the monitoring of treatment response (Fig 5).

Our investigation unveiled the perturbation of crucial gene sets in response to *Mtb* infection. The gene expression profile in individuals with tuberculosis (TB) predominantly revolves around the activation of defense responses to bacterial and viral infections [20]. Numerous comparative studies have been undertaken to explore various biomarkers or biomarker combinations for the diagnosis of tuberculosis [21–27]. The Type-II interferon signaling pathway is very well-explored which is crucial for both innate and adaptive immunity against viral, bacterial, and protozoan infections. Within this pathway, IFN-gamma (IFN-γ) is primarily secreted by various immune cells such as natural killer (NK) cells, macrophages, and T-cells, typically in response to IL-12 and IL-18 [28]. Guanylate binding proteins (GBPs), including GBP5, function as hydrolases and are induced by IFN. They play a vital role in regulating host innate immune responses, notably by eliciting the host apoptosis processes during pathogenic infection [29, 30]. Previous studies have reported elevated whole blood transcriptional levels of GBP5 in active TB [17, 27, 31]. The upregulation of the GBP5 protein in whole blood samples of active TB was initially reported by Yao et al. [32]. It is worth noting that one of the GBPs, GBP1, has a protective role during TB infection [33], and its expression levels showed a correlation with the GBP5, BATF2, and EPSTI1, aligning with our findings [34].

IFN-γ triggers the JAK-STAT pathway, leading to the activation of IRF1 and AIM2. IRF1 promotes the expression of IFNs and GBPs, such as GBP5, which permeabilize the *Mtb* membrane, releasing *Mtb* DNA and other components. AIM2 detects intracellular DNA, facilitating the release of pro-inflammatory cytokines IL-18 and IL-1β, which have been associated with protection against pulmonary tuberculosis (PTB) [35]. *Mtb* infection also triggers the NFκB signaling pathway, regulating the expression of IL-10 and other GBPs, and can activate ASC-1, leading to plasma membrane lysis for cytokine release and mediating cellular Pyroptosis [36]. Our study revealed a strong correlation between the upregulation of GBP5 and the high expression levels of innate immune response proteins like GBP2, EIF2AK2, IFITM3, and IFIT2. This finding further underscores the potential role of GBP5 in innate immunity processes, particularly in the activation of the AIM2 inflammasome during tuberculosis infection.

The IFITM family of proteins encodes three transmembrane proteins, specifically IFITM1, IFITM2, and IFITM3, which are widely recognized as interferon (IFN)-induced transmembrane (IFITM) genes. These genes are known to be stimulated by various proinflammatory cytokines including IL-6, IL-1β, IFNβ, TNF, especially within the context of TLR2/4 signaling pathways and in response to *Mtb* infection. These genes have been demonstrated to limit the intracellular growth of *Mtb* [37]. In *Mtb-*infected monocytes, IFITM3 is observed to co-localize with *Mtb* within maturing phagosome, contributing to the enhancement of endosomal acidification. The overexpression of IFITM3 has a significant inhibitory effect on *Mtb* growth [38]. Several biomarkers GBP1, GBP2, GBP5, STAT1, IFIT3, and IFITM3, identified and validated in this study are found to be crucial components of TB diagnostic panels in other research as highly valuable elements of their TB diagnostic markers [10, 25, 39]. In addition, an interesting study emphasized the significance of a 4-gene transcriptional signature, which includes GBP1, ID3, P2RY14, and IFITM3 [40]. Another study showed a distinct 3-gene transcriptional signature, consisting of GBP5, DUSP3, and KLF2 [17], serves as the basis for TB diagnostic tests.

EPSTI1 (epithelial stromal interaction 1) is an interferon (IFN) inducible gene [41] which has been found as a stromal fibroblast-induced gene in breast cancer and also highly upregulated in invasive breast carcinomas as compared to normal breast [42]. EPSTI1 is involved in the regulation of apoptotic pathways via physical interactions with the apoptotic initiator Caspase-8 and also with AKT1 and BCAR3 [43]. In the current study, the EPSTI1 gene was found to be upregulated which suggests that increased levels of EPSTI1 may increase Caspase-8 levels and potentially modulate apoptotic pathways through alternative mechanisms, such as by interacting with AKT1 or BCAR3 proteins.

One other important protein, BATF2, identified in our study, is a member of the activator protein-1 (AP-1) transcription factor family. It is induced by interferon (IFN) in mononuclear phagocytic cells and has been demonstrated to upregulate in response to innate immune stimulation, triggered by factors like lipopolysaccharide (LPS) or *Mtb*. BATF2 can bind with IRF1 (IFN regulatory factor 1), promoting the activation of downstream elements, some of which are also a part of the host immune response against *Mtb* [44, 45]. Although systemic IFN activity is well-established in TB [46], the high expression of BATF2 is likely a result of IFN responses rather than direct *Mtb* stimulation of circulating blood cells. A study also reported that BATF2 gene expression served as a unique blood transcript capable of accurately distinguishing individuals with active from those with latent tuberculosis (LTBI) and healthy individuals [47]. Another upregulated gene in TB patient samples is TAP1, which plays a role in peptide transport for antigen presentation. It forms a complex with MHC class-I molecules [48], influencing the activation of cytotoxic T-cells. Zak et al., reported GBP1 as a signature of disease risk wherein GBP1, STAT1, and TAP1 were found to have a protective role during TB infection and were associated with favorable clinical outcomes [33].

## Conclusions

In summary, our comprehensive analysis of a gene network highlights perturbed gene biosignatures triggered by *Mtb* infection and their changes during TB treatment. GBP5, IFITM3, and EPSTI1 showed significant perturbations in response to TB infection, rendering them potential targets for molecular drug interventions in TB infection. GBP5 demonstrates a substantial diagnostic utility and holds promise as a prime biomarker candidate for the development of transcriptome-based TB prognostic or diagnostic assays.

## Supporting information

S1 Fig Functional and expression analysis of 18 genes of Module-1. (A) Gene Ontology (GO) analysis uncovers the genes functionally related to interferon-gamma signaling during TB infection; (B) The network of these Module-1 genes was constructed using the STRING database, providing insights into their interactions and relationships (C) and (D) features Heatmap showing the expression of 18 genes within Module, derived from dataset GSE42826 and GSE42830 respectively. Each row in the Heatmaps corresponds to an individual gene.
(PDF)

S2 Fig Heatmap representing the expression validation of 8 important genes in GSE31348 dataset during TB treatment at 5 different time points. (PDF)

S1 Table Two Modules were identified by the MCODE app in Cytoscape. (PDF)

S2 Table The functions of the common top 8 proteins selected based on T-test/ ANOVA and MCODE score greater than equal to 10. (PDF)
