# Correction: Computational reassessment of RNA-seq data reveals key genes in active tuberculosis

**DOI:** 10.1371/journal.pone.0319694

**Published:** 2025-03-04

**Authors:** Rakesh Arya, Hemlata Shakya, Reetika Chaurasia, Surendra Kumar, Joseph M. Vinetz, Jong Joo Kim

There are errors in the Author Contributions. The correct contributions are:

**Conceptualization:** Rakesh Arya, Hemlata Shakya

**Data curation:** Rakesh Arya, Hemlata Shakya

**Formal analysis:** Rakesh Arya, Hemlata Shakya

**Investigation:** Rakesh Arya

**Methodology:** Rakesh Arya

**Project administration:** Jong Joo Kim

**Resources:** Jong Joo Kim

**Software:** Rakesh Arya

**Supervision:** Jong Joo Kim

**Validation:** Rakesh Arya

**Visualization:** Rakesh Arya

**Writing – original draft:** Rakesh Arya, Hemlata Shakya, Reetika Chaurasia.

**Writing – review and editing:** Rakesh Arya, Hemlata Shakya, Surendra Kumar, Reetika Chaurasia, Joseph M. Vinetz.

## References

[1] Arya R, Shakya H, Chaurasia R, Kumar S, Vinetz JM, Kim JJ. Computational reassessment of RNA-seq data reveals key genes in active tuberculosis. PLoS One. 2024;19(6):e0305582. doi: 10.1371/journal.pone.0305582 38935691 PMC11210783

