# Supplementary material for: Computational reassessment of RNA-seq data reveals key genes in active tuberculosis
Source: PLoS One. 2024 Jun 27;19(6):e0305582. doi: 10.1371/journal.pone.0305582 (PMC11210783; doi:10.1371/journal.pone.0305582)
Supplement: S1 Fig — (A) Gene Ontology (GO) analysis uncovers the genes functionally related to interferon-gamma signaling during TB infection; (B) The network of these Module-1 genes was constructed using the STRING database, providing insights into their interactions and relationships (C) and (D) features Heatmap showing the expression of 18 genes within Module, derived from dataset GSE42826 and GSE42830 respectively. Each row in the Heatmaps corresponds to an individual gene. (PDF) [file pone.0305582.s001.pdf]

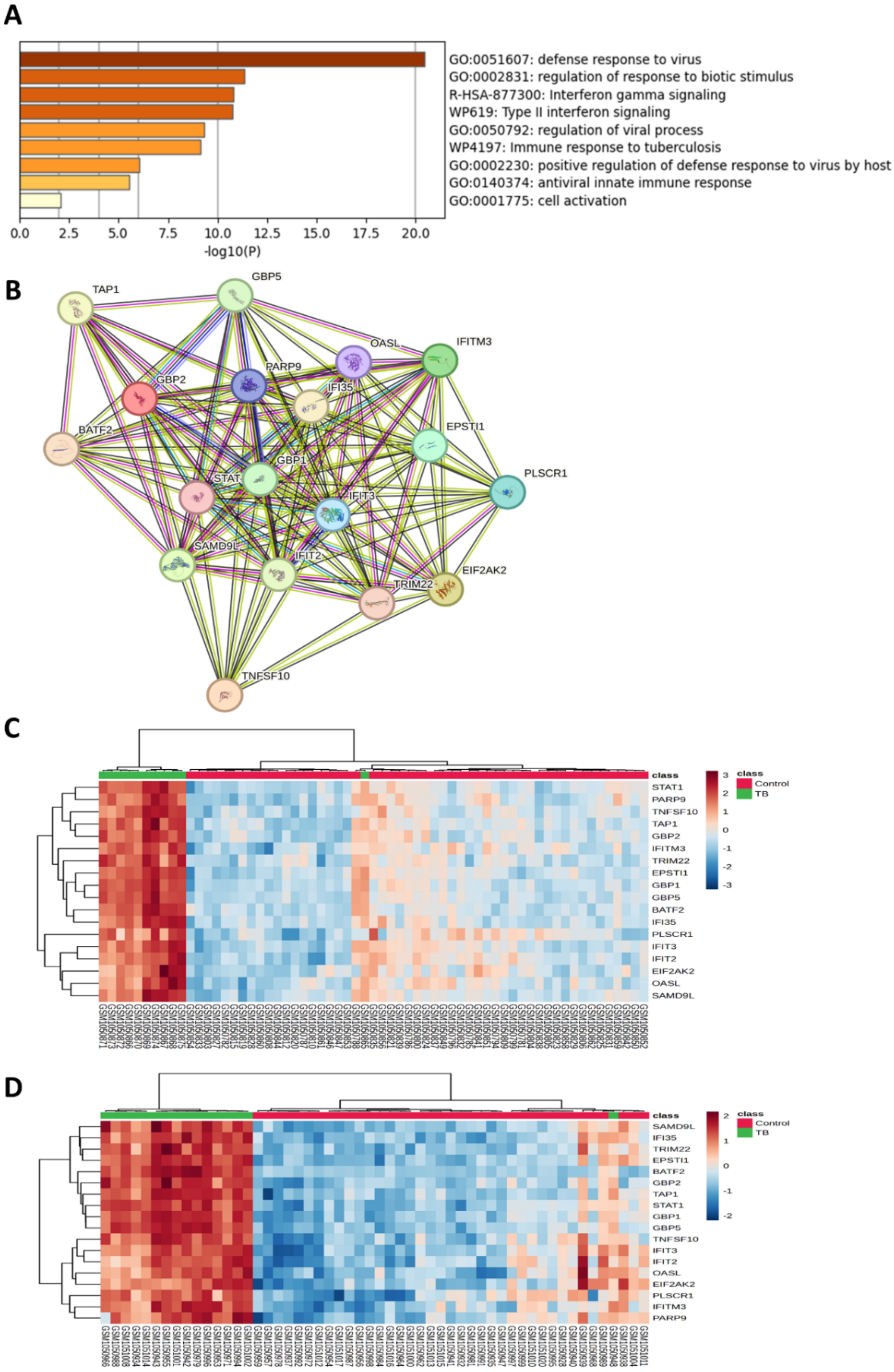
A
GO:0051607: defense response to virus
GO:0002831: regulation of response to biotic stimulus
R-HSA-877300: Interferon gamma signaling
WP619: Type II interferon signaling
GO:0050792: regulation of viral process
WP4197: Immune response to tuberculosis
GO:0002230: positive regulation of defense response to virus by host
GO:0140374: antiviral innate immune response
GO:0001775: cell activation
0.0 2.5 5.0 7.5 10.0 12.5 15.0 17.5 20.0
-log10(P)
B
TAP1
GBP5
OASL
IFITM3
PARP9
GBP2
IFI35
BATF2
EPSTI1
GBP1
PLSCR1
STAT
IFIT3
SAMD9L
IFIT2
EIF2AK2
TRIM22
TNFSF10
C
class
STAT1
PARP9
TNFSF10
TAP1
GBP2
IFITM3
TRIM22
EPSTI1
GBP1
GBP5
BATF2
IFI35
PLSCR1
IFIT3
IFIT2
EIF2AK2
OASL
SAMD9L
3 2 1 0 -1 -2 -3
class
Control
TB
D
class
SAMD9L
IFI35
TRIM22
EPSTI1
BATF2
GBP2
TAP1
STAT1
GBP1
GBP5
TNFSF10
IFIT3
IFIT2
OASL
EIF2AK2
PLSCR1
IFITM3
PARP9
2 1 0 -1 -2
class
Control
TB

S1 Fig. Functional and expression analysis of 18 genes of Module-1.

(A) Gene Ontology (GO) analysis uncovers the genes functionally related to interferon-gamma signaling during TB infection; (B) The network of these Module-1 genes was constructed using the STRING database, providing insights into their interactions and relationships (C) and (D) features Heatmap showing the expression of 18 genes within Module, derived from dataset GSE42826 and GSE42830 respectively. Each row in the Heatmaps corresponds to an individual gene.
