# Supplementary material for: Computational reassessment of RNA-seq data reveals key genes in active tuberculosis
Source: PLoS One. 2024 Jun 27;19(6):e0305582. doi: 10.1371/journal.pone.0305582 (PMC11210783; doi:10.1371/journal.pone.0305582)
Supplement: S2 Fig — (PDF) [file pone.0305582.s002.pdf]

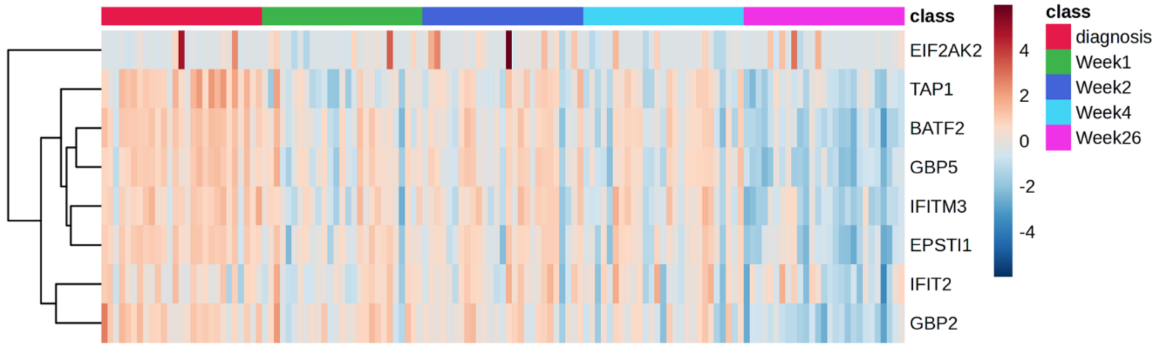

S2 Fig. Heatmap representing the expression validation of 8 important genes in GSE31348 dataset during TB treatment at five different time points.
