# Supplementary material for: Computational reassessment of RNA-seq data reveals key genes in active tuberculosis
Source: PLoS One. 2024 Jun 27;19(6):e0305582. doi: 10.1371/journal.pone.0305582 (PMC11210783; doi:10.1371/journal.pone.0305582)
Supplement: S1 Table — (PDF) [file pone.0305582.s003.pdf]

S1 Table. Two Modules were identified by the MCODE app in Cytoscape.

**Modules**	**Gene ID**
Module-1 (18 genes)	OASL, STAT1, BATF2, GBP1, IFIT3, EIF2AK2, IFIT2, PLSCR1. IFI35, TAP1, GBP2, TRIM22, SAMD9L, TNFSF10, IFITM3, PARP9, GBP5, EPSTI1
Module-2 (3 genes)	FCGR1A, FCGR1B, SERPING1
