# Supplementary material for: Computational reassessment of RNA-seq data reveals key genes in active tuberculosis
Source: PLoS One. 2024 Jun 27;19(6):e0305582. doi: 10.1371/journal.pone.0305582 (PMC11210783; doi:10.1371/journal.pone.0305582)
Supplement: S2 Table — (PDF) [file pone.0305582.s004.pdf]

S2 Table. The functions of the common top 8 proteins selected based on T-test/ANOVA and MCODE score ≥10.

Gene ID	MCODE Score	Protein Name	Functions (from UniProt database)
EIF2AK2	12	Interferon-induced, double-stranded RNA-activated protein kinase/ eukaryotic translation initiation factor 2 alpha kinase 2	IFN-induced dsRNA-dependent serine/threonine-protein kinase that phosphorylates the alpha subunit of eukaryotic translation initiation factor 2 (EIF2S1/eIF-2-alpha) and plays a key role in the innate immune response to viral infection. Either as an adapter protein and/or via its kinase activity, can regulate various signaling pathways (p38 MAP kinase, NF-kappa-B and insulin signaling pathways) and transcription factors (JUN, STAT1, STAT3, IRF1, ATF3) involved in the expression of genes encoding pro-inflammatory cytokines and IFNs
GBP2	12	Guanylate-binding protein 2	Interferon (IFN)-inducible GTPase that plays important roles in innate immunity against a diverse range of bacterial, viral and protozoan pathogens. Induced by IFN-gamma during macrophage activation, and by TNF and IL1B
GBP5	12	Guanylate-binding protein 5	Interferon (IFN)-inducible GTPase that plays important roles in innate immunity against a diverse range of bacterial, viral and protozoan pathogens. Induced by IFN-gamma in endothelial cells and in LPS-primed macrophages
IFIT2	11.54	Interferon-induced protein with	IFN-induced antiviral protein which inhibits expression of viral messenger RNAs lacking

		tetratricopeptide repeats 2	2'-O-methylation of the 5' cap. The ribose 2'-O-methylation would provide a molecular signature to distinguish between self and non-self mRNAs by the host during viral infection
IFITM3	11.54	Interferon-induced transmembrane protein 3	IFN-induced antiviral protein which disrupts intracellular cholesterol homeostasis. Inhibits the entry of viruses to the host cell cytoplasm by preventing viral fusion with cholesterol depleted endosomes
EPSTI1	11.54	Epithelial-stromal interaction protein 1	Plays a role in M1 macrophage polarization and is required for the proper regulation of gene expression during M1 versus M2 macrophage differentiation
BATF2	11	Basic leucine zipper transcriptional factor ATF-like 2	AP-1 family transcription factor that controls the differentiation of lineage-specific cells in the immune system. Also known as Suppressor of AP-1 regulated by IFN. Following infection, participates in the differentiation of CD8+ thymic conventional dendritic cells in the immune system
TAP1	10	Antigen peptide transporter 1/ transporter 1, ATP binding cassette subfamily B member	Typically transports intracellular peptide antigens of 8 to 13 amino acids that arise from cytosolic proteolysis via IFNG-induced immunoproteasome. Upregulated by IFN-gamma
